# Effect of Deformation on the Corrosion Behavior of Friction Stir Welded Joints of 2024 Aluminum Alloy

**DOI:** 10.3390/ma15062157

**Published:** 2022-03-16

**Authors:** Qiu Pang, Man Zhao, Zhi-Li Hu

**Affiliations:** 1 Department of Mechanical and Electrical Engineering, Wuhan Donghu University, Wuhan 430212, China; pqiuhit@126.com; 2 Hubei Key Laboratory of Advanced Technology for Automotive Components, Wuhan University of Technology, Wuhan 430070, China; manzhao0112@163.com; 3 Hubei Collaborative Innovation Center for Automotive Components Technology, Wuhan University of Technology, Wuhan 430070, China

**Keywords:** 2024 aluminum alloy, plastic deformation, friction stir welded joint, corrosion behavior

## Abstract

Friction stir welding (FSW) of aluminum alloys is an advanced manufacturing technology to realize lightweight bodywork. However, most studies only focus on the mechanical properties and corrosion behaviors of the welded joints. The effect of deformation on the corrosion behavior of FSWed joints is unclear. In this work, the plastic deformation behavior was characterized using uniaxial tensile tests. The effect of deformation on the corrosion behavior of a 2024 aluminum alloy nugget was studied by using a Tafel polarization curve, electrochemical impedance spectroscopy, exfoliation corrosion test, scanning electron microscopy and energy dispersive spectrometer, and transmission electron microscopy. The results show that the corrosion resistance of FSWed joints with different deformation degrees can be ranked as: 0% > 7% > 10% > 4%, and an “inflection point” appears at 7%. The corrosion potential and current density at 7% are near the values at 0%, and the 7% sample shows less corrosion rate than all other deformation samples. Only pitting and bubbling occur in the sample in 96 h. With an increase in plastic deformation, the dislocations and dislocation rings increase, there is an increase in the surrounding winding precipitates. The impurity phase is cleaved by dislocations; a reduction in the size of the impurity phase with low chemical activity can be observed, resulting in an increase in corrosion resistance. However, the transgranular and intergranular cracks appear on the 10% deformation sample. They almost always develop along the grain boundaries after initiation, making them more susceptible to corrosion.

## 1. Introduction

With the rapid development of the automobile industry, the demands of energy savings and emission reductions are increasingly urgent, which make lightweight vehicles an irreversible trend in the automobile industry. Tailor-welded forming is considered to be an advanced manufacturing technology to realize lightweight metal bodies. It is a technology that connects several metal plates of different materials and different sizes together and then makes a large deformation to form complex shapes, which can achieve a double weight reduction effect of the materials and structures [1]. At present, the welding forming technology of steel has been widely used in automobiles. However, due to quality problems of traditional fusion welding of aluminum alloys, it is easy to produce defects such as pores, hot cracks and slag inclusions, resulting in poor formability, which cannot be used for aluminum tailor-welded forming. As the most widely used alloy in the aerospace industry, 2024 aluminum alloy is used to manufacture the main stress components of aircraft, aircraft structures (skin, skeleton, rib beam, spacer frame, etc.), rivets, missile components, truck hub, propeller components and other structural components [2]. 2024 aluminum alloy is widely used in aircraft manufacturing, marine structures and automotive applications due to its low density, good corrosion resistance, high formability and bonding ability, and high specific strength [3]. Therefore, the dissimilar welding process of these alloys has a wide range of applications in different manufacturing disciplines. Aluminum alloys are usually classified as “refractory” using conventional fusion welding methods. This is due to defects that occur during solidification, such as loss of alloying elements, pores and intermetallic compounds [4]. FSW is a solid metal joining technology, and the mechanical properties of joints have obvious advantages over traditional fusion welding. Hu et al. found that the tensile strength and yield strength were significantly improved of 2024 aluminum alloy FSWed joints after severe plastic deformation. And the 2.88 mm 2024 aluminum alloy FSWed joint had better thermal stability and high ductility during the solution treatment at 450~495 °C [5,6]. Therefore, FSW is widely used in the aerospace industry, especially Al-Cu (2xxx series) and Al-Zn (7xxx series). The corrosion resistance is an important performance that affects its safety, life and other aspects. It is a hot spot of current research. In 2001, Zucchi et al. compared the corrosion behavior of FSWed joints and MIG welded joints of AA 5083, and the results showed that FSWed joints did not easily produce stress corrosion cracking (SCC) in two kinds of test solutions (3.5% NaCl + 0.3 g/L H_2_O and exfoliation corrosion (EXCO) solution), whereas MIG joints cracked in them [7]. In 2004, Squillace et al. welded AA2024-T3 aluminum alloy with FSW and TIG methods. The polarization curves and EIS tests showed that the base metal of the two joints had an obvious pitting tendency, while the nugget zone (NZ) and heat-affected zone (HAZ) of FSW exhibited passive behavior for pitting [8]. In 2012, Huang et al. designed a new FSW tool composed of rolling balls, namely, in-situ rolling friction stir welding (IRSW) for welding 2219 aluminum alloy. The test results showed that IRSW reduced the residual tensile stress and improved the corrosion resistance [9].

In addition to the above welding of the same kind of materials, the welding of dissimilar materials is also very common. For example, in the research on friction stir welding of dissimilar aluminum, Davoodi et al. found that the corrosion resistance of the FSW region was between AA5083 and AA7023. Corrosion started at the FSW boundary and the area around the intermetallic particles, especially on the AA7023 side [10]. Lakshminarayanan et al. used a multi-criterion optimization procedure for multi-seam friction stir clad joints of dissimilar magnesium-aluminum alloys to obtain higher interface strength and lower corrosion rate [11]. In addition to the above butt welding, there are also forms of lap welding. Gharavi et al. issued that the weld zone of AA6061-T6 FSW lap joints was more prone to intergranular corrosion and pitting corrosion than the base metal in the immersion corrosion test [12]. In the welding process, scholars at home and abroad constantly change the welding process parameters for research and exploration. Some studies showed that AA 7075-AA 6056 FSWed joints had little effect on SCC at low strain rates in 3.5% NaCl solution, while AA 2219 FSWed joints showed better stress corrosion cracking resistance at strain rates of 10-6s-1 and 10-7s-1 [13]. Rambabu et al. established a mathematical model of the corrosion resistance of a friction stir welded AA 2219 aluminum alloy by incorporating process parameters and optimized the model with simulated annealing optimization technology to maximize the corrosion resistance of the friction stir welded AA 2219 aluminum alloy joints [14]. Sinhmar et al. used seven different speed combinations to carry out friction stir welding of an AA2014 aluminum alloy and found that it showed high microhardness and corrosion resistance at low rotation speed and high traverse speed [15]. 

In addition, the corrosion resistance was also greatly influenced by the corrosion medium. Yun et al. carried out a cyclic neutral salt spray test on the friction stir welding of an A7075-A6N01 aluminum alloy and found that the HAZ boundary, TMAZ (thermomechanical affected zone) boundary and its surrounding areas were most prone to corrosion [16]. Fu et al. found that the corrosion resistance of the FSWed joint of 7075 aluminum alloy was lower than that of the pure aluminum clad-alloy in an acidic continuous salt spray test. Corrosion started from pitting and intergranular corrosion and finally evolved into EXCO [17]. Xu et al. showed the corrosion resistance of 2219-O aluminum alloy FSWed joints. It was found that the top had the best corrosion resistance compared to the bottom and base material [18]. Li et al. immersed TMAZ and NZ of 2024-T3 aluminum alloy FSW sheets in three different corrosion solutions (3.5 wt% NaCl, 3.5 wt% NaCl + 1 wt% HCl and EXCO), and the results indicated that TMAZ and SZ of the FSWed joints presented different corrosion performances. Moreover, the NZ corrosion resistance was higher than TMAZ [19]. Jayaraj et al. studied the corrosion resistance of SZ of FSWed dissimilar joints of AA6061 Al alloy and AZ31B Mg alloy. The corrosion rate was higher in acidic media than in alkaline and neutral media [20]. Protonr et al. showed that the nugget was sensitive to both intergranular and intragranular corrosion in a 2050 aluminum alloy FSWed joint [21]. Niu et al. investigated the effect of base metal (BM) locations on the corrosion behavior of friction stir welded dissimilar 2024-T351 to 7075-T651 aluminum alloys joints in an EXCO solution. The corrosion resistance of SZs was similar to that of the BM positioning in the retreating side (RS), and intergranular corrosion was still the main mechanism [22]. Zhang et al. analyzed the influence of different welding heat inputs on the corrosion behavior of 7050 aluminum alloy FSWed joints and found that they were highly sensitive to stress corrosion. Whether optimizing welding parameters or changing heat input, it was difficult to improve the stress corrosion sensitivity by controlling the welding process itself [23]. However, these are the mechanical properties and corrosion behaviors of the as-welded state. For tailor-welded forming, these laws cannot be applied because FSWed joints have experienced large plastic deformation. The effect of deformation on the corrosion behavior of FSWed joints is not clear. Therefore, this work presents the different plastic deformations of welded joints determined by uniaxial tensile tests. On this basis, the corrosion resistance is analyzed by optical microscope (OM), scanning electron microscopy and energy dispersive spectrometer (SEM/EDS) and transmission electron microscopy (TEM).

## 2. Materials and Methods

### 2.1. Material and Sample Preparation

**Figure 1:**
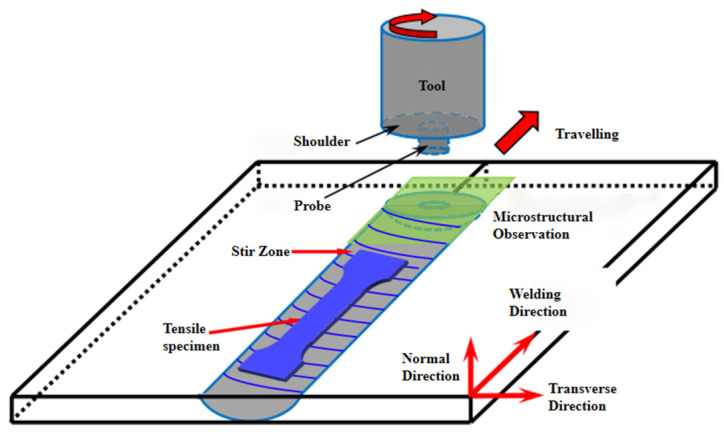
The orientation of the 3D model of the tensile sample.

The base metal of the test is a 2024-O aluminum alloy, and the size is 300 × 80 × 3 mm. The 2024 aluminum alloy is butt-welded using the FSW process at a rotation speed of 800 rpm and a welding speed of 100 mm/min. A conical high alloy steel mixing head with the right thread is selected. The shaft shoulder diameter is 10 mm, the length is 3 mm, and the inclination angle is 3°. The welding direction is perpendicular to the rolling direction of the base metal. The chemical composition of the base metal is shown in Table 1. According to GB/T 288.1-2010, the size of the tensile sample is selected, and the width of the weld nugget zone is 6 mm, as shown in Figure 1. 

The sheared samples are subjected to room temperature uniaxial tensile tests on the WDW-100 universal testing machine at a speed of 2 mm/min. The plastic deformation is 0%, 4%, 7%, and 10%, respectively.

### 2.2. Electrochemical Experiment

The corrosion resistance of the sample was tested by a Parstat 2273 electrochemical workstation (Princeton MC, Princeton, NJ, USA). Four groups of experiments were performed in a battery electrode of 3.5% NaCl solution (25 ± 1 °C) with 0%, 4%, 7%, and 10%, respectively. The working electrode was the upper surface of the joint, and the scanning speed was 10 mV/s by using three-electrode dynamic potential scanning. Before the experiment, the samples were cut into 6 × 6 × 3 mm. The workfaces to be tested were ground with sandpaper to be bright and flat, then washed and dried with alcohol. The non-workfaces were sealed with epoxy resin to ensure that the final test area was 0.36 cm^2^.

The open circuit potential (OCP) was a relatively stable corrosion potential for each sample after being immersed in the 3.5% NaCl solution for 1.5 h. The Tafel polarization curve (Tafel curve) and electrochemical impedance spectroscopy (EIS) were measured. The selection range of the polarization curve was—0.2~+0.5 V with a scan rate of 0.167 mV/s. In the EIS test, the amplitude of the AC signal was ±10 mV, and the frequency was 0.01~105 Hz.

### 2.3. Exfoliation Corrosion Experiment

The EXCO experiment was conducted according to GB/T 22639-2008. The ratio of the solution was 236 g (2.16 g/mL) NaCl + 50 g (2.10 g/mL) KNO_3_ + 6.3 mL (1.40 g/mL) HNO_3_, diluted to 1000 mL with distilled water, sealed with rosin on the nonworking surface. The samples were soaked at a temperature of 25 ± 2 °C and a humidity of 45 ± 6% for different times and then taken out. The corrosion morphology was analyzed by metallographic optical microscope (OM) and scanning electron microscopy (SEM).

### 2.4. Microstructural Investigation

Prior to each observation, samples were subjected to rough grinding, fine grinding, polishing, cleaning and drying. Microstructural changes were examined using SEM (model: Zeiss Ultra Plus, resolution: 1.0 nm, max voltage: 15 kV, Zeiss, Oberkochen, Germany). Before TEM imaging (model: JEM-2100F, resolution: 0.23 nm, accelerating voltage: 180 kV, Nippon Electronics Co., Ltd, Tokyo, Japan), the samples were ground to 0.05–0.08 mm by fine grinding and then thinned to 0.06 mm with a diameter of 3 mm. A Struers TenuPol-5 electrolytic double spray instrument (electrolyte: 20% HNO_3_ +80% CH_3_OH, voltage: 25 V, Struers, Denmark) was used to reduce the thickness of the double spray. 

## 3. Results and Discussion

### 3.1. Corrosion Behavior

**Figure 2:**
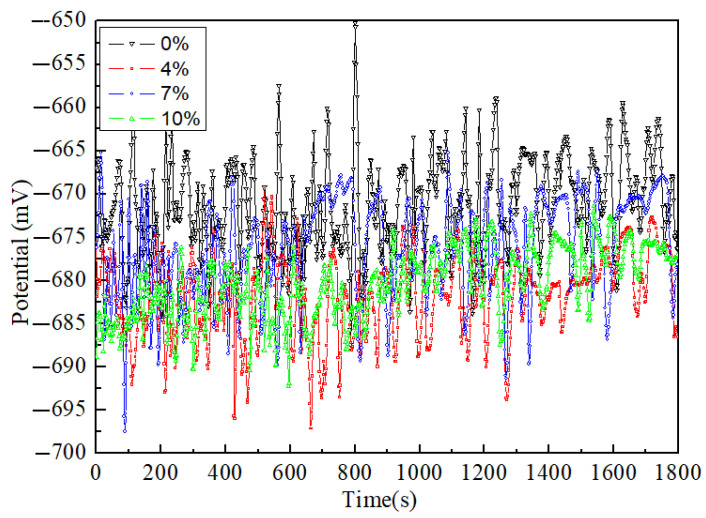
Open circuit potential of the 2024 FSWed joints with different deformations (0%, 4%, 7%, and 10%) in the 3.5% NaCl electrolyte.

Apart from the changes in corrosion current (Icorr) and potential (Ecorr), the polarization curves differ markedly in appearance and structure, especially on the anodic branch. In fact, the presence of anode branches represents to some extent the formation of a passivation layer. The anode step then means attenuation and destruction of the passivation layer [24]. The relatively stable OCPs of the 0%, 4%, 7% and 10% samples in the 3.5% NaCl electrolyte for 1.5 h are shown in Figure 2. After immersing each sample into the corrosive media, there is a meaningful difference among the initial OCP values. Each sample shows an increase in the potential value as an enhancement result of the passivation layer [25]. The passivation layer dissolves continuously, and the surface charge moves continuously, which leads to a sharp increase in the OCP at 700 s. The sample gradually stabilizes after 900 s. The OCP of FSW joints fluctuates within ±5–10 mV and tends to be stable after 600–1200 s. The higher the OCP, the smaller the corrosion tendency and the better the corrosion resistance is. It is obvious that the average OCP at 0% is higher than those of the other three deformed samples (4%, 7%, and 10%), which indicates that the corrosion resistance at 0% is the best. Among the three deformation values, only the OCP at 7% is close to 0%, with the second-best corrosion resistance, whereas 4% is the lowest and has the worst corrosion resistance. This is different from the traditional corrosion performance of aluminum alloy, which is generally steady state, would be a change of 5 mV in OCP over a 10 min period [26]. This is mainly because the microstructure of aluminum alloy after FSW is more complex, and the microstructure uniformity is worse than that of base metal, resulting in a large fluctuation of OCP.

**Figure 3:**
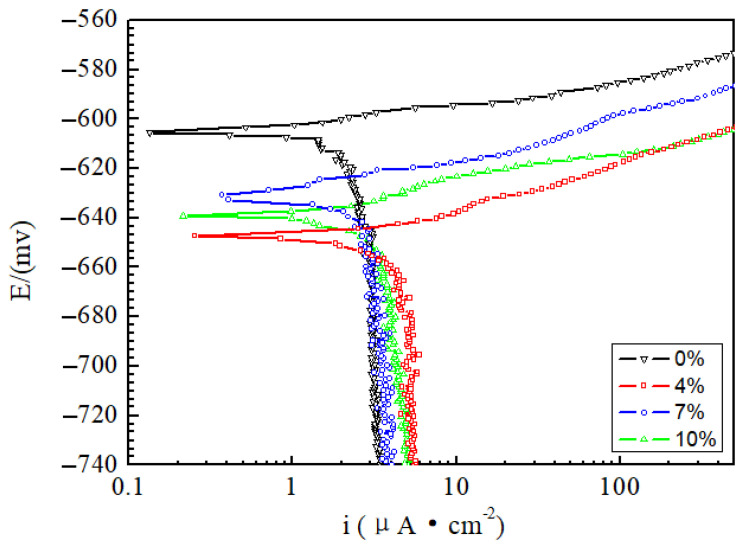
Tafel polarization curves of the 2024 FSWed joints with different deformations (0%, 4%, 7%, and 10%) in the 3.5% NaCl electrolyte.

**Figure 4:**
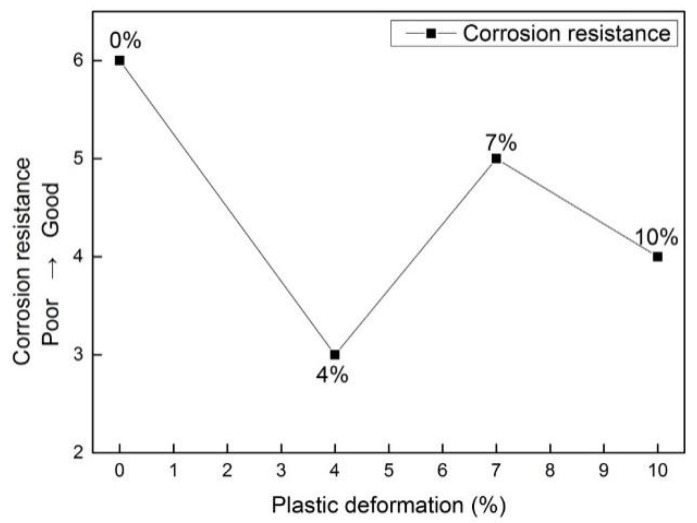
Corrosion resistance trend with different plastic deformations (0%, 4%, 7%, 10%).

The results of the Tafel curves are displayed in Figure 3, and the extracted data is presented in Table 2. The corrosion potential and corrosion current density reflect the thermodynamic and kinetic tendency of the sample, respectively, which reflect the corrosion performance together. Therefore, the corrosion current density (i), corrosion potential (E) and Tafel slopes are also reported. According to the reference [27], the reference electrode (RE) is a saturated calomel electrode. The more negative the corrosion potential and the higher the corrosion current density, the greater the corrosion tendency of the sample. As shown, corrosion potential values are similar to the OCP results in Figure 2. For all the potential values presented vs. RE, the corrosion potential at 0% is higher than that of the other three deformation samples, and the current density is the smallest. Therefore, the corrosion resistance is the best. The corrosion potential at 4% is the smallest. Its current density is twice as high as 0%, so its corrosion resistance is the worst. With an increase in deformation, the corrosion resistance increases at 7%, and its corrosion potential and current density are near the values at 0%. Furthermore, an “inflection point” occurs at 7%, and the corrosion resistance is near that of the undeformed (0%), as shown in Figure 4. These four samples show the same dynamic behavior, i.e., a diffusion-controlled behavior is observed in the cathodic branch since the oxygen is involved in the following reaction [28]:

O_2_ + 4H_2_O + 4e^−^ = 4OH^−^ (1)

The breakdown at 4% occurs at high potential in the anodic branch because the current density exceeds 10%. The overall dynamic behavior is relatively active, including the cathode.

**Figure 5:**
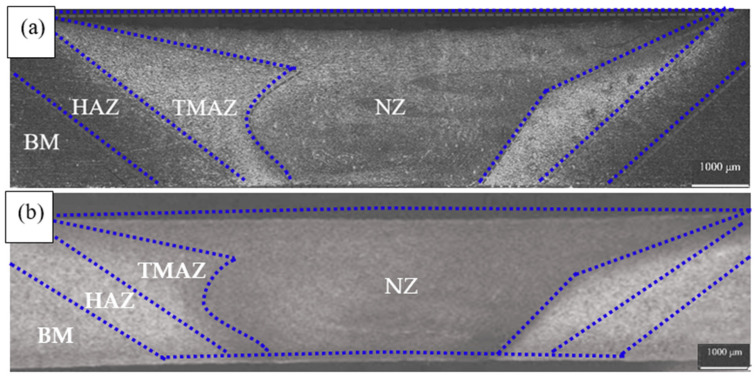
The optical micrograph of welding zones after deformation (**a**) 0%, (**b**) 10%.

In addition, the micromorphology of the weld area with different deformations is observed in Figure 5, and the results show that they are in all four areas, which can be divided into nugget zone (NZ), thermomechanical affected zone (TMAZ), heat affected zone (HAZ) and base metal (BM) [29]. There is little difference before and after deformation, as shown in Figure 5, slight necking at 10% deformation. Dynamic recrystallization and fine equiaxed grains are formed in the nugget area of a welded joint due to the strong stirring and crushing of the stirring needle and the action of friction heat. The grain size of the material in the middle of the welded member is larger than that at the bottom of the welded member in Figure 5. In contrast to conventional fusion welding, FSW does not involve melt. The joining of similar or dissimilar metals and alloys in FSW is achieved by extreme deformation creating dynamic recrystallization (DRX), which permits flow in the solid-state by sliding between recrystallized, equiaxed, and usually submicron grains in the weld zone.

**Figure 6:**
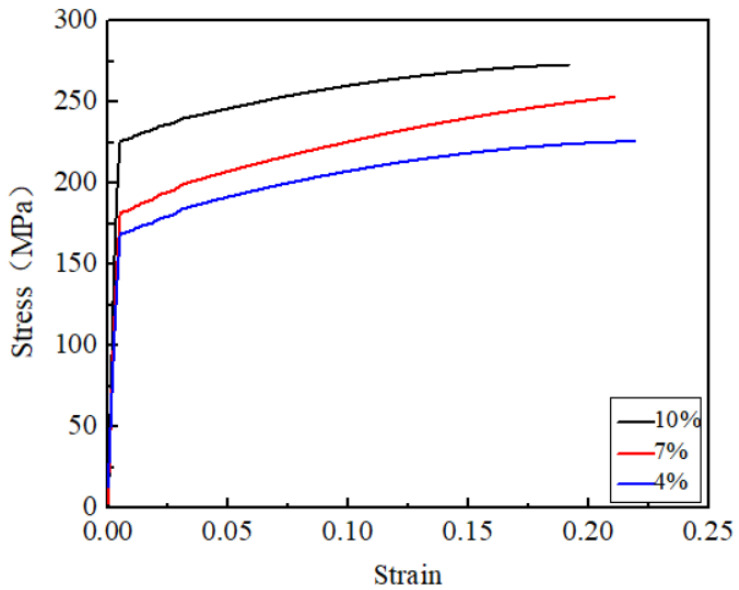
The stress-strain curves of the samples (4%, 7%, 10%).

The stress-strain curves of the samples are shown in Figure 6. At the beginning of the tensile strength test, the stress of the joint increases rapidly with the increase in tensile strain. Then, the increase in stress tends to be gentle. This is mainly due to the dynamic softening caused by work hardening, dynamic recovery and dynamic recrystallization, as well as the damage evolution process. 

**Figure 7:**
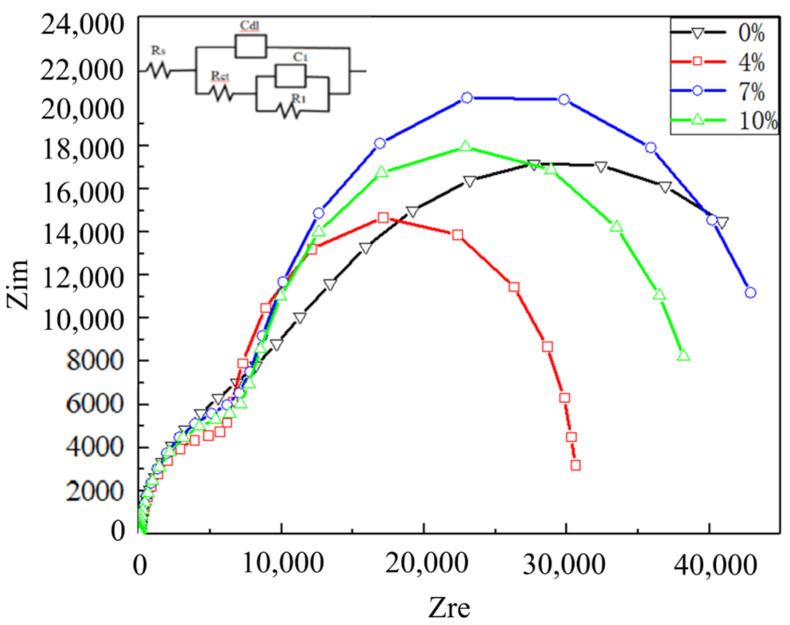
Nyquist results from the EIS test of the 2024 FSWed joints with different deformations in the 3.5% NaCl electrolyte.

**Figure 8:**
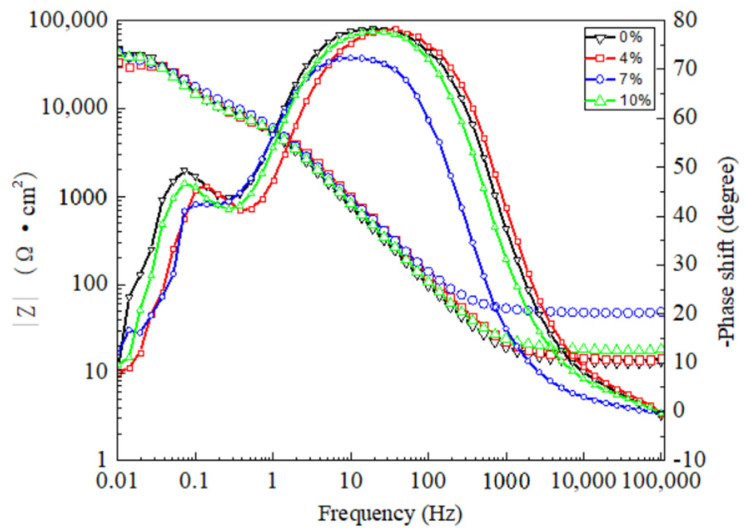
Bode diagram results from the EIS test of the 2024 FSWed joints with different deformations in the 3.5% NaCl electrolyte.

The EIS results as Nyquist and Bode phase data are shown in Figure 7 and Figure 8. An equivalent circuit diagram is inserted to fit the EIS, where RS and RCT represent the solution resistance and electrode surface charge transfer resistance, respectively, and Cdl represents the charge and discharge process on the electrode surface instead of an equivalent capacitance [30]. The analysis is carried out strictly according to the circuit diagram when the equivalent circuit diagram is used to fit all the measured points. According to the EIS results and the characteristics of the 2024 aluminum alloy, C1 and R1 represent the equivalent capacitance and equivalent resistance formed by the oxide film on the surface of the nugget, respectively. A constant-phase element Q (Q1, Q2) is chosen with a nonlinear capacitor to obtain an optimal solution by fitting. Combined with Table 3, the impedance can be calculated by using the following formula [31]: when the equivalent circuit diagram is used to fit all the measured points, the analysis is carried out strictly according to the circuit diagram. All samples are carried out in the same 3.5 wt% NaCl.

(2) $$Z=\frac{1}{Y_{0}}{\left(jw\right)}^{-n}$$

Here *Y*_0_ and *n* represent the respective admittance and dispersion index in the fitting process. The whole equivalent circuit impedance *Z_F_* is shown in Formula (3):

(3) ZF=Rct+1(1R1+jwC1)

The oxide layer has a large impedance value, while the capacitor layer is a small isolation layer. It can be seen from Table 3 that they are not at the same level because the impedance value is far greater than the capacitance value. Therefore, *C*_1_ on the impedance can be ignored, and *Z_F_* can be replaced by Formula (4) [32]:

(4) $$Z_{F}\approx R_{ct}+R_{1}$$

In Table 3, the impedance at 0%, 4%, 7% and 10% can be calculated as 52.63, 30.70, 45.99 and 39.85, respectively (unit: kΩ∙cm^−2^). The greater the impedance, the better the corrosion resistance of the material. The impedance at 0% exceeds 1.7 times that at 4%. The corrosion resistance at 0% is the best, 7% is the second-best, and 4% is the worst. This also corresponds to the previous OCP and Tafel results. 

### 3.2. Exfoliation Corrosion

**Figure 9:**
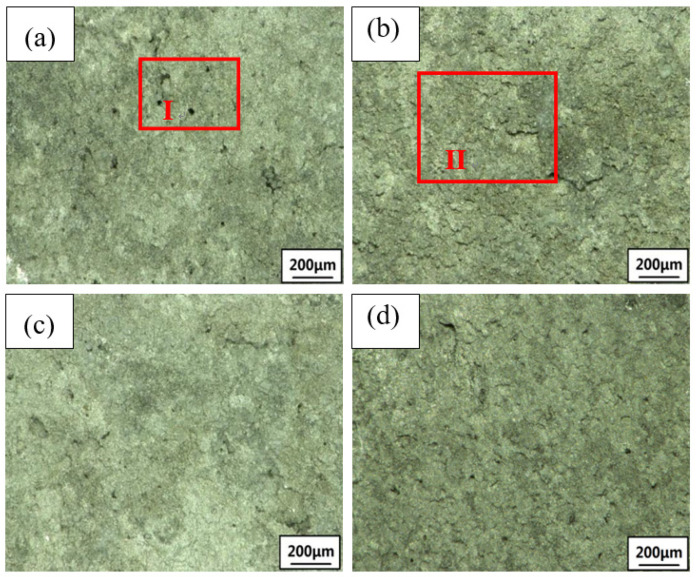
Corrosion morphology of the 2024 FSWed joints with different deformations after 24 h (**a**) 0%, (**b**) 4%, (**c**) 7%, (**d**) 10%.

**Figure 10:**
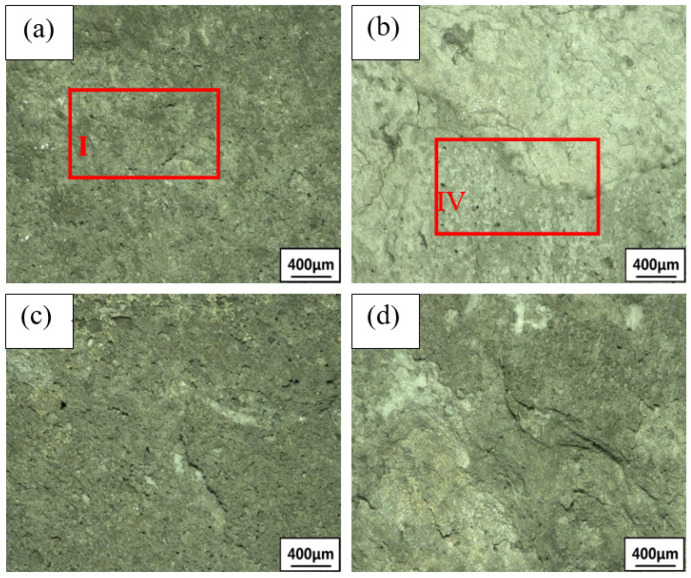
Corrosion morphology of the 2024 FSWed joints with different deformations after 48 h (**a**) 0%, (**b**) 4%, (**c**) 7%, (**d**) 10%.

**Figure 11:**
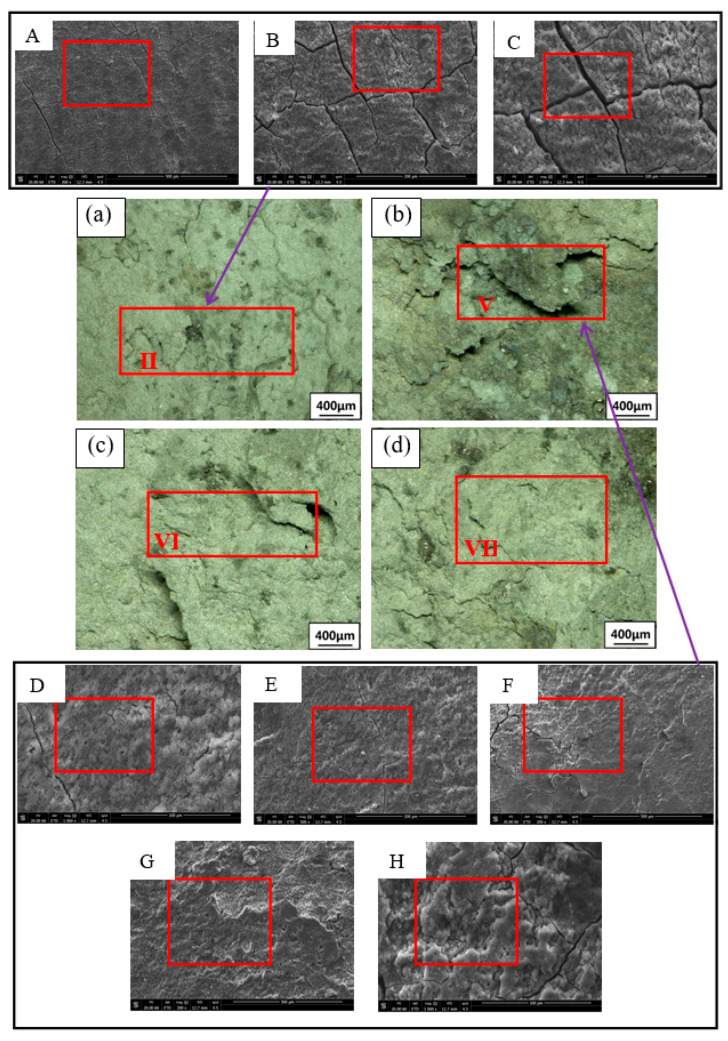
Corrosion morphology of the 2024 FSWed joints with different deformations after 96 h (**a**) 0%, (**b**) 4%, (**c**) 7%, (**d**) 10%, **A** is pitting corrosion in 0% sample, **B** is bubbles in 0% sample, **C** is cracking in 0% sample, **D** is pitting corrosion in 4% sample, **E** is bubbles in 4% sample, **F** is cracking in 4% sample, **G** is delamination in 4% sample, **H** is spalling in 4% sample.

Due to the activity of the Mg element in the 2×××series aluminum alloy, it dissolves preferentially and corrodes as an anode in exfoliation corrosion solution, forming large pits. Then, intergranular corrosion or exfoliation corrosion occurs slowly. The OM and SEM images of the corrosion are shown in Figure 9, Figure 10 and Figure 11. The samples are immersed in the same peeling corrosion solution for 24 h, 48 h and 96 h, respectively. Generally, with an extension of corrosion time, the sample surfaces undergo processes such as corrosion pitting, corrosion bubbling, corrosion cracking, corrosion stratification and surface spalling [33]. With the extension of corrosion time, the 0% sample surface demonstrated pitting corrosion at 24 h and 48 h (region I in Figure 9a and Figure 10a. If the corrosion time increases to 96 h, in combination with OM and SEM, 0% of the sample surface showed pitting corrosion (Figure 11A), bubbles (Figure 11B) and a small part of corrosion cracking (Figure 11C) at 96 h, resulting in minimal corrosion. However, the 4% sample has pitting and stratification within 24 h (region III in Figure 9b). When the corrosion time increases to 48 h, the corrosion stratification area increases, even producing partial spalling (region IV in Figure 10b). When the time is extended to 96 h, 4% of the samples have pitting corrosion (Figure 11D), bubbles (Figure 11E) and cracking (Figure 11F) within 96 h, the corrosion delamination area increases, and a large number of corrosion products will be produced on the surface, accompanied by serious corrosion delamination (Figure 11G) and spalling (Figure 11H). Delamination and spalling occur alternately, resulting in multilayer spalling. There is no doubt that the corrosion degree of 4% is the most serious in the whole process. The 7% sample shows less corrosion rate than all other deformation samples. Only pitting and bubbling occur in the sample in 96 h, and even progress from cracking to stratification, but all the pieces of material do not fall off (region VI in Figure 11c). The 10% sample also show stratification, and some of them peel off at 96 h (region VII in Figure 11d). Although their peeling times are later than at 4%, there are relatively fewer corrosion products than at 4%. In summary, the corrosion degree of undeformed (0%) samples is the lightest, followed by 7% and 10%, the 4% sample has the most severe exfoliation corrosion, which is consistent with the previous electrochemical test.

**Figure 12:**
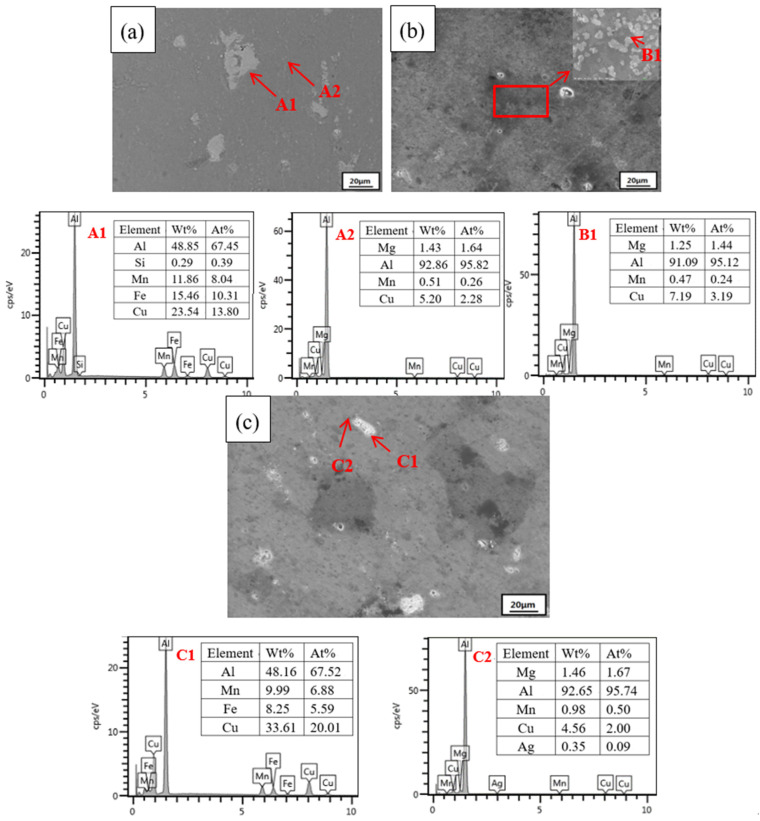
SEM images and EDS results of the 2024 FSWed joints second phase with different deformations (**a**) 0%, (**b**) 4%, (**c**) 7%, **A1** is welding nugget of 0% sample, **A2** is second phases in 0% sample, **B1** is second phases in 4% sample, **C1** is welding nugget of 7% sample, **C2** is second phases in 7% sample.

Figure 12 shows the SEM images of 2024 FSWed joints with different plastic deformations. After the nuggets undergo large plastic deformation and high-temperature changes during FSW, obvious dynamic recrystallization occurs in the microstructure, leading to small grains [34]. As shown in A1 of Figure 12a, there are more coarse precipitated phases in the 0% deformation sample. These coarse precipitated phases are impurities that are rich in copper, iron and manganese, as observed through EDS (A1 pointed by the red arrow), which occupy a large position in the 2024 welding nugget. Similarly, in the 4% and 7% SEM images, the large precipitated phases are still impurities rich in copper, iron and manganese by EDS (C1 pointed by the red arrow). However, with the appearance of plastic deformation, the impurity phase is cleaved by dislocation. Compared with the coarse impurity phase at 0% sample, their sizes are greatly reduced and occupy a small position in the 2024 welding nugget. Most of these impurities exist as elements with low chemical activity, making them difficult to corrode.

From the SEM images, it is not difficult to find that the impurity phase at 0% is more than five times that of the deformed sample morphology. The dynamic property of corrosion is weak, so the corrosion resistance is good. Outside of the impurity phase, the other second phases (A2, B1, C2 pointed by red arrows) have also been tested by EDS. They all contain alloy elements such as copper, magnesium, and manganese, which conform to the composition of the 2024 precipitated phase. There is no essential difference between the undeformed sample and the deformed sample for the above precipitated phase. In summary, with the emergence of plastic deformation, the impurity phase is cleaved by dislocation, resulting in a decrease in the size of the impurity phase containing low chemical activity. While the impurity phase of the 0% sample is still large, the relatively difficult corrosion area is also very large, so the corrosion resistance is improved, which is better than that of the deformed samples.

**Figure 13:**
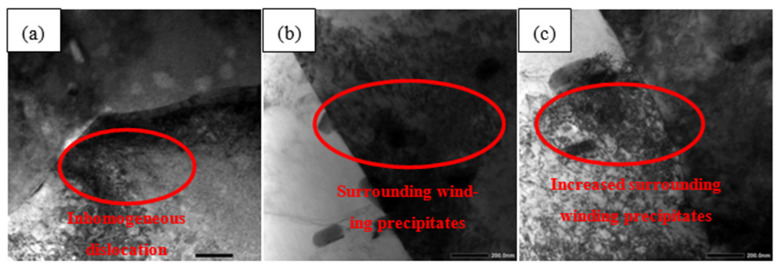
Dislocation morphology of the 2024 FSWed joints with different deformations (**a**) 4%, (**b**) 7%, (**c**) 10%.

There are a large number of dislocation and dislocation rings in the matrix because plastic deformation can promote the aggregation and slippage of dislocations in empty space [35]. With the increase in plastic deformation, dislocations and dislocation rings increase and surrounding winding precipitates increase, which suppresses corrosion. As shown in Figure 13a, the dislocation at 4% is relatively small because of the small plastic deformation, and the areas where dislocations appear are distributed. In addition, some locations have almost no dislocations (red region in Figure 13a). Furthermore, surrounding winding precipitates will only appear where there is dislocation. Compared with 4%, the dislocation distribution at 7% is relatively homogeneous, and the overall density is increased in Figure 12b. Moreover, the number of entangled precipitated phases is small (red region in Figure 13b).

In Figure 13c, the dislocation distribution at 10% is the densest and very homogeneous, so the number of precipitated phases wrapped around it also increases (red region in Figure 13c). On the one hand, there is no difference in the precipitated phases of various plastic deformations in the above SEM. On the other hand, the dislocation at 4% is non-uniform. Therefore, the phase surrounded by 4% is the least, which leads to the most corrosion phases (the element of Mg in the precipitated phase is preferentially corroded because of its high chemical activity). According to the number and distribution of dislocations mentioned above, the corrosion resistance from the highest to the lowest should be: 10% > 7% > 4%. However, the transgranular and intergranular cracks appear on the 10% deformation sample. They almost always develop along the grain boundaries after initiation, making them more susceptible to corrosion. Therefore, the corrosion resistance at 10% is reduced. The final corrosion resistance from the highest to the lowest is as follows: 7% > 10% > 4%.

## 4. Conclusions

(1) With plastic deformations of 0%, 4%, 7% and 10%, the corrosion resistance of 2024 aluminum alloy FSWed joints from the highest to the lowest is as follows: 0% > 7% > 10% > 4%. The greater the impedance and the OCP, the better the corrosion resistance of the material. Especially, the corrosion potential and current density at 7% are near the values at 0%. An “inflection point” occurs at 7%. Corrosion starts from pitting corrosion, goes through blistering, cracking, delamination and stripping, and finally develops into spalling corrosion; Among them, the 4% deformed samples had delamination after soaking for 24 h, and the 0% deformed samples had cracking for the first time after soaking for 96 h.
(2) The open circuit potential of each sample tends to be stable after 900 s. The average open circuit potential from high to low is 0% > 7% > 10% > 4%; In a Tafel curve, the absolute value of self-corrosion potential of the 4% deformed sample is the largest, which is 648 mV, which is higher than that of the other three deformed samples, and the corrosion current density is 4.63 μA·cm^−2^, which is basically twice that of 0% deformed samples, and the corrosion resistance is the worst. In EIS analysis, the electrochemical impedance values of 0%, 4%, 7% and 10% deformed samples are 52.63, 30.70, 45.99 and 39.85 (unit: KΩ∙cm^−2^) respectively. Based on the above test data, the corrosion resistance of the sample from high to low is 0% > 7% > 10% > 4%.
(3) For the 0% deformation sample, the precipitated phases are impurities that are rich in copper, iron and manganese in the 2024 welding nugget. Most of these impurities exist as elements with low chemical activity, making them difficult to corrode. With the appearance of plastic deformation, the impurity phase is cleaved by dislocations, resulting in a reduction in the size of the impurity phase with low chemical activity. With an increase in plastic deformation, the dislocations and dislocation rings increase, resulting in an increase in corrosion resistance. However, the transgranular and intergranular cracks appear on the 10% deformation sample. They almost always develop along the grain boundaries after initiation, making them more susceptible to corrosion.

## Figures and Tables

**Table 1:** Chemical composition of the 2024 aluminum alloy plates (wt%).

Cu	Mg	Mn	Fe	Si	Cr	Zn	Ti	Other	Al
3.8~4.9	1.2~1.8	0.3~0.9	≤0.5	≤0.5	≤0.1	≤0.25	≤0.15	≤0.15	Bal.

**Table 2:** Data extracted from the Tafel polarization curves in Figure 3.

Deformation (%)	E (mV)	i (μA∙cm^−2^)	Intercept |a|	Slope |b|
0	−605	2.48	248	931
4	−648	4.63	59	922
7	−630	2.88	279	789
10	−639	3.85	142	895

**Table 3:** EIS data combined with the equivalent circuit diagram in Figure 7.

Deformation (%)	R_s_ (Ω∙cm^−2^)	Q_1_-Y_0_ × 10^−5^ (Ω^−1^S^n^)	Q_1_-n	R_ct_ (kΩ∙cm^−2^)	Q_2_-Y_0_ × 10^−5^ (Ω^−1^S^n^)	Q_2_-n	R_1_ (kΩ∙cm^−2^)
0	17.88	2.671	0.89	13.15	10.14	0.8	39.48
4	14.28	2.265	0.909	10.52	10.3	1.19	20.18
7	13.27	2.919	0.909	12.72	12.7	1.08	33.27
10	18.17	2.766	0.908	12.04	14.12	1.11	27.81

## Data Availability

Data sharing not applicable. No new data were created or analyzed in this study. Data sharing is not applicable to this article.
